# Immune checkpoint inhibitors in melanoma: mechanisms, immune cell interactions, and the tumour microenvironment

**DOI:** 10.3389/fimmu.2025.1691608

**Published:** 2025-11-19

**Authors:** Yiwen Liang, Yan Zheng, Yuyan Zeng, Chengjing Hu, Yuqi Si, Xiqian Fan, Qihua Chen

**Affiliations:** 1 Medical School, Hunan University of Chinese Medicine, Changsha, Hunan, China; 2 School of Acupuncture-moxibustion, Tuina and Rehabilitation, Hunan University of Chinese Medicine, Changsha, Hunan, China; 3 College of Integrated Chinese and Western Medicine, Hunan University of Traditional Chinese Medicine, Changsha, Hunan, China; 4 School of traditional Chinese Medicine, Hunan University of Chinese Medicine, Changsha, Hunan, China; 5 School of Public Health, Chengdu University of Chinese Medicine, Chengdu, Sichuan, China; 6 Department of Surgery for Male Disease of First Hospital, Hunan University of Chinese Medicine, Changsha, Hunan, China

**Keywords:** melanoma, immune checkpoint inhibitors, immune cells, tumor microenvironment, adjuvant therapy, neoadjuvant therapy, tumor staging, tissue-resident memory T cells

## Abstract

Melanoma is a highly aggressive and metastatic malignant tumor originating from melanocytes, with globally rising incidence rates that pose significant challenges to patient prognosis. Traditional therapies for advanced melanoma have limited efficacy. In recent years, the emergence of immune checkpoint inhibitors (ICIs) has significantly altered this landscape by reactivating the body’s antitumor immune response through blocking interactions between immune checkpoint proteins and their ligands, demonstrating remarkable therapeutic outcomes. However, some patients do not respond to ICIs or develop resistance, indicating that treatment responses involve complex interactions between tumors, immune cells, and the tumor microenvironment. This review comprehensively summarizes the mechanisms of ICIs, delves into the roles of various immune cells (including T cells, NK cells, macrophages, T helper cells, dendritic cells, and B cells) and the tumor microenvironment (TME), and explores their impact on ICI efficacy. It further distinguishes the application of ICBs across different disease stages (primary, adjuvant, neoadjuvant, and metastatic) and highlights the role of skin-specific immune cells (e.g., TRM, Langerhans cells) and microenvironmental components (e.g., skin microbiome). This review focuses on the mechanisms of ICIs in melanoma therapy, exploring the interactions between immune cells and the skin microenvironment in melanoma development and their impact on ICI efficacy. It aims to provide new insights and theoretical foundations for optimizing immunotherapy strategies in melanoma treatment.

## 1 Introduction

Melanoma, a malignant neoplasm originating from melanocytes, has shown an alarming growth trend globally (1). Statistics indicate a continuous rise in incidence over the past several decades, positioning it as one of the major diseases that seriously threaten human health. In China, although the incidence of melanoma is relatively low at approximately 0.9 per 100,000 individuals, due to the large population base, the number of new cases annually is substantial, reaching around 20,000, with a persistently high mortality rate, underscoring its significant hazard (2). Characterized by high invasiveness and metastatic potential, melanoma tends to develop distant metastases, leading to poor patient prognosis and a low 5-year survival rate. Traditional therapies such as surgical resection, chemotherapy, and radiotherapy have limited efficacy in advanced melanoma, failing to meet clinical needs (3).

In recent years, the advent of immunotherapy, particularly the application of immune checkpoint inhibitors (ICIs), has brought about revolutionary advancements in melanoma treatment. Immune checkpoints are critical regulatory mechanisms within the immune system that normally maintain immune homeostasis and prevent excessive immune responses from damaging self-tissues (4). However, tumor cells can exploit this mechanism by upregulating the expression of immune checkpoint molecules, thereby inhibiting the immune system’s recognition and attack on tumor cells and achieving immune evasion. The mechanism of ICIs lies in blocking the interaction between immune checkpoint proteins and their ligands, releasing the inhibition imposed by tumor cells on the immune system, and reactivating the body’s antitumor immune response (5). In melanoma therapy, ICIs have demonstrated remarkable efficacy, significantly extending patient survival and improving quality of life. For instance, inhibitors targeting programmed death-1 (PD-1) and its ligand (PD-L1), as well as cytotoxic T-lymphocyte-associated antigen 4 (CTLA-4), have been widely applied clinically and achieved notable therapeutic outcomes (6–8). Nevertheless, despite the impressive achievements of ICIs in melanoma treatment, not all patients benefit, and some experience resistance or immune-related adverse events during treatment (9). This indicates that the development and response to ICIs in melanoma involve a highly complex process, encompassing multidimensional interactions between tumor cells, immune cells, and the tumor microenvironment.

The tumor microenvironment plays a crucial role as the site for tumor cell growth, proliferation, and metastasis in the pathogenesis and progression of melanoma. For cutaneous melanoma, the skin microenvironment represents the initial and critical TME niche. The TME encompasses various cellular components such as fibroblasts, endothelial cells, macrophages, mast cells, and biological active molecules like the extracellular matrix, cytokines, and chemokines (10). These components interact with tumor cells through direct contact or cytokine secretion, forming a complex network that collectively regulates the biological behavior of tumor cells, including proliferation, invasion, metastasis, and immune evasion (11). A thorough investigation into the mechanisms of interaction between immune cells and the TME in melanoma pathogenesis and progression is essential for enhancing our understanding of melanoma etiology and optimizing immunotherapy strategies.

This review aims to comprehensively summarize the mechanisms of ICIs in melanoma treatment, delve into the interactions between immune cells and the tumor microenvironment in the context of melanoma development across different stages, and examine how these interactions influence the efficacy of ICI treatment. It seeks to provide new insights and theoretical foundations for the clinical treatment of melanoma.

## 2 Overview of melanoma

### 2.1 Definition and characteristics

**Figure 1:**
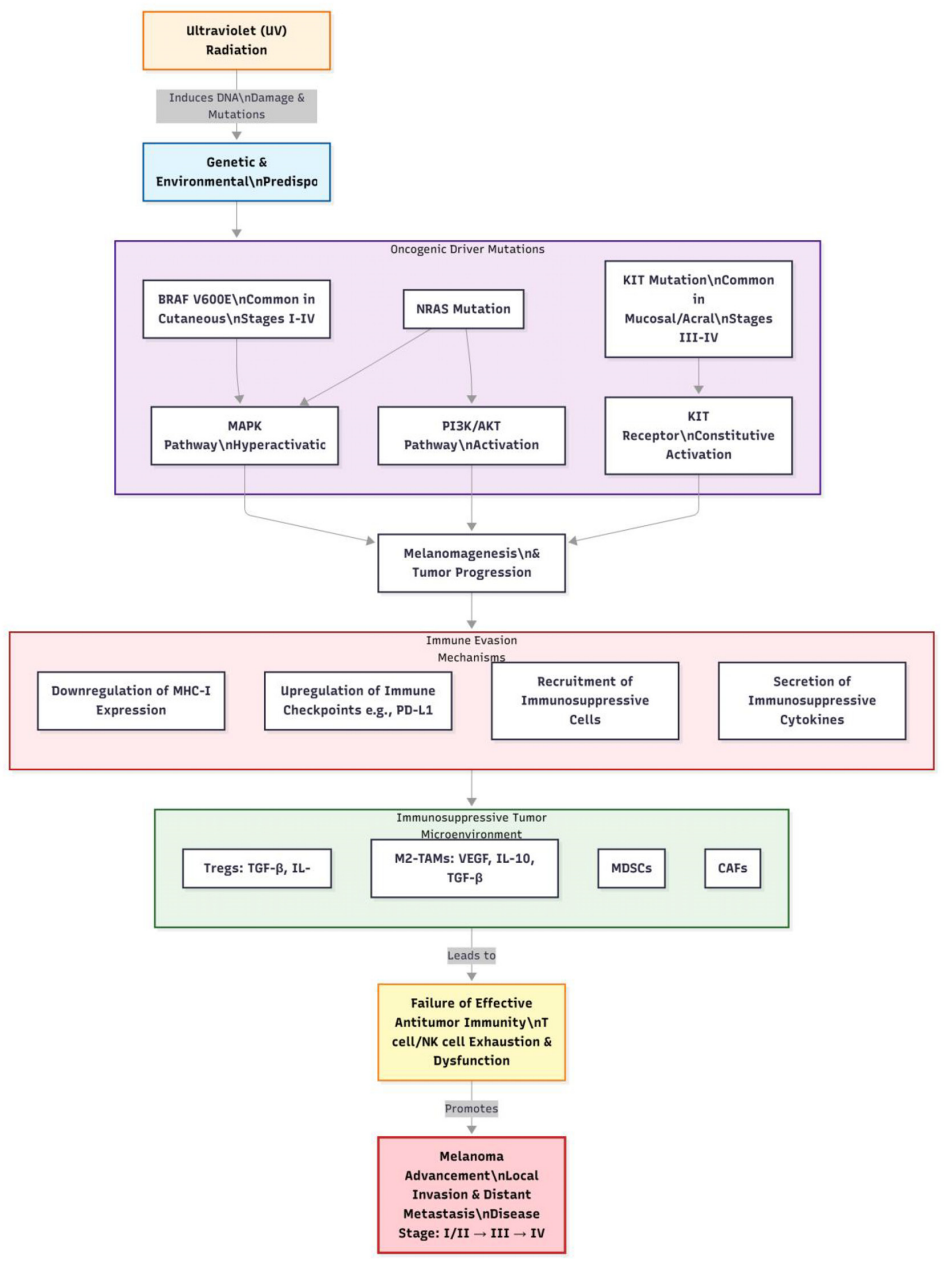
The pathogenesis of melanoma.

Melanoma is a highly malignant tumor originating from melanocytes, most commonly occurring in the skin but also found in mucosal surfaces, the uveal tract of the eye, and other locations. It is characterized by significant invasiveness and metastatic potential, key factors contributing to poor patient prognosis. Melanoma cells can breach the basement membrane, invade surrounding tissues, and disseminate to distant organs via the lymphatic and circulatory systems; common sites of metastasis include lymph nodes, lungs, liver, bones, and brain (12). Once metastasis occurs, the 5-year survival rate declines sharply (13). Clinically, melanoma often manifests as changes in pre-existing moles, such as rapid enlargement, irregular shape, uneven coloration, indistinct borders, surface ulceration, itching, or bleeding. Additionally, some melanomas may appear *de novo* without obvious predisposing factors. These characteristics complicate early diagnosis, leading to potential neglect by patients and delayed treatment. The complex pathogenesis of melanoma, integrating genetic, environmental, and immunological factors, is visually summarized in **Figure 1**. Melanoma is staged based on the American Joint Committee on Cancer (AJCC) TNM system, which classifies the disease into stages I to IV. Stage I and II represent localized primary tumors with varying thickness and ulceration status. Stage III indicates regional metastasis to lymph nodes or in-transit metastases. Stage IV signifies distant metastasis to organs like the lungs, liver, or brain. Prognosis varies significantly by stage, with 5-year survival rates exceeding 80% for stages I-II, approximately 50% for stage III, and dropping below 20% for stage IV (13). This staging is crucial for guiding treatment decisions, including the use of ICIs in adjuvant or metastatic settings.

### 2.2 Pathogenesis

The pathogenesis of melanoma is complex, involving genetic, environmental, and immunological factors. From a genetic perspective, approximately 10% of melanoma cases exhibit familial inheritance. Research indicates that mutations in multiple genes are closely associated with melanoma development, including BRAF, NRAS, and KIT (14–16). The V600E mutation in the BRAF gene is the most prevalent, accounting for 80%-90% of all BRAF mutations, which leads to continuous activation of the mitogen-activated protein kinase (MAPK) signaling pathway, promoting abnormal proliferation and transformation of melanocytes (17, 18). Mutations in the NRAS gene activate downstream phosphatidylinositol-3-kinase (PI3K)/protein kinase B (AKT) signaling pathways, influencing cell growth, survival, and metabolism. Mutations in the KIT gene are more frequently observed in mucosal, acral, and chronically sun-damaged melanomas, leading to sustained activation of the KIT receptor and promoting tumor cell proliferation and survival (19). Notably, BRAF V600E mutations are most common in cutaneous melanomas (across stages I-IV), while KIT mutations are more prevalent in mucosal or acral melanomas (often presenting at later stages).

Environmental factors play a critical role in the pathogenesis of melanoma, with ultraviolet (UV) radiation being the most established risk factor. UV radiation induces DNA damage, leading to gene mutations. Specifically, UVB radiation (280–320 nm) can directly damage DNA pyrimidine bases, forming cyclobutane pyrimidine dimers (CPDs) and 6–4 photoproducts (6-4PP). If these damages are not promptly repaired, they can lead to gene mutations and subsequently cause melanoma (19). Moreover, prolonged sun exposure, history of sunburns, and frequent outdoor activities increase the risk of developing melanoma.

**Figure 2:**
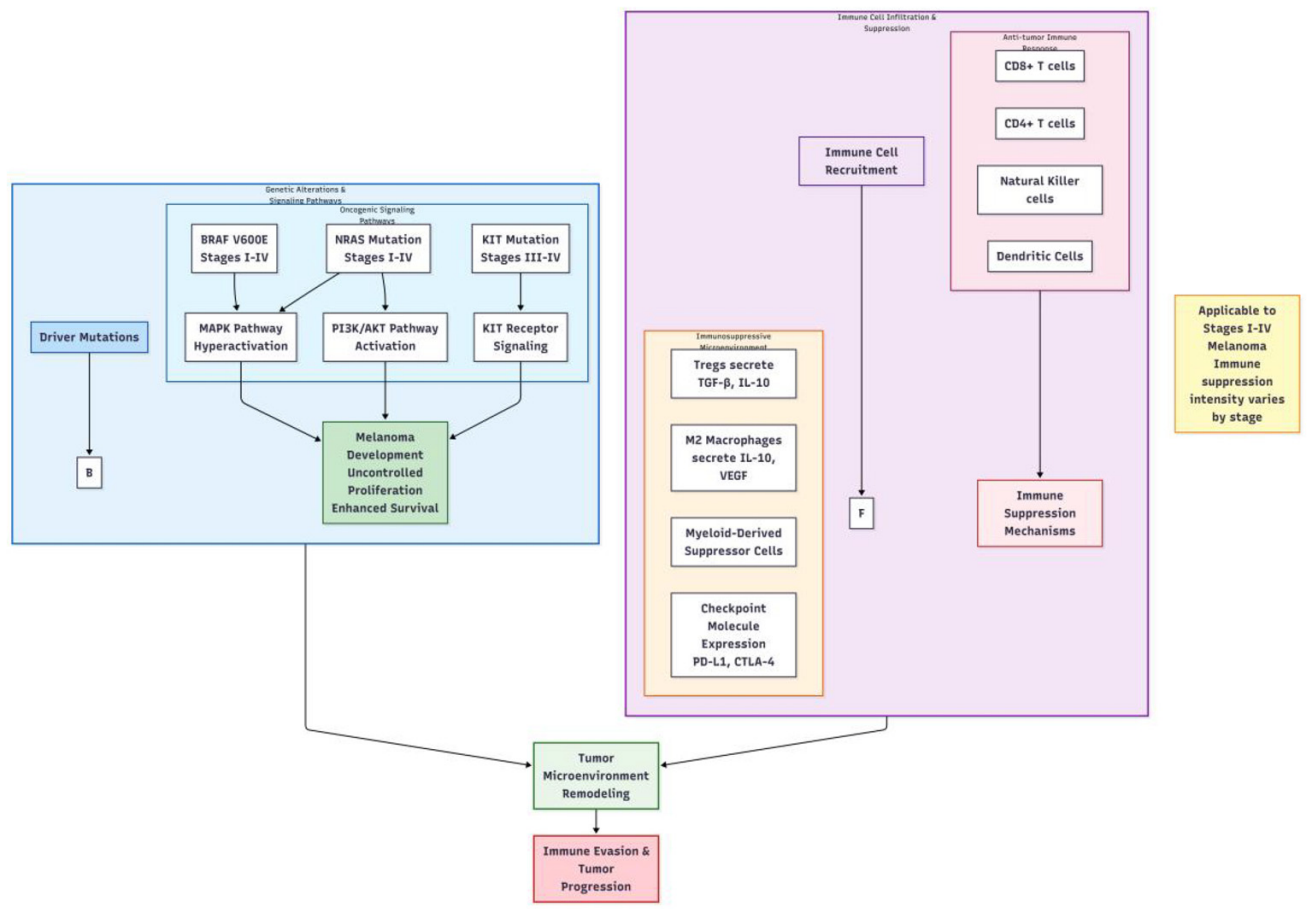
Key genetic alterations and immune cell infiltration in melanoma.

Immune evasion is a crucial aspect of melanoma progression. Tumor cells can employ various mechanisms to escape immune surveillance and attack (20, 21). One mechanism involves downregulating the expression of major histocompatibility complex (MHC) class I molecules, reducing the efficiency of antigen presentation and making it difficult for cytotoxic T lymphocytes (CTLs) to recognize tumor cells (22). Another mechanism includes secreting immunosuppressive factors such as transforming growth factor-beta (TGF-β) and interleukin-10 (IL-10), which inhibit the activity and function of immune cells (23). Furthermore, immunosuppressive cells within the tumor microenvironment, such as regulatory T cells (Tregs) and myeloid-derived suppressor cells (MDSCs), can suppress antitumor immune responses through multiple pathways, providing favorable conditions for tumor growth and metastasis (24). The interplay between key genetic alterations and immune cell infiltration mechanisms in melanoma pathogenesis is comprehensively depicted in **Figure 2**.

## 3 Immune checkpoint inhibitors

### 3.1 Common types

Immune checkpoint inhibitors are a category of therapeutic agents that modulate the function of the immune system for cancer treatment. Their primary mechanism of action involves blocking the interaction between immune checkpoint proteins and their ligands, thereby releasing the inhibition exerted by tumor cells on the immune system. Currently, widely used immune checkpoint inhibitors in clinical settings primarily include monoclonal antibodies targeting programmed death receptor 1 (PD-1), programmed death-ligand 1 (PD-L1), and cytotoxic T-lymphocyte-associated antigen 4 (CTLA-4) (25). Pembrolizumab and nivolumab represent two common PD-1 inhibitors. Pembrolizumab, marketed under the name Keytruda, has received approval from the U.S. Food and Drug Administration (FDA) for the treatment of various cancers, such as melanoma, non-small cell lung carcinoma (NSCLC), and head and neck squamous cell carcinoma. It is approved for adjuvant treatment of stage III melanoma and for metastatic (stage IV) disease. Nivolumab, known by the trade name Opdivo, also demonstrates efficacy in treating melanoma, lung carcinoma, renal carcinoma, and other tumors (26). It is similarly approved for adjuvant (stage III) and metastatic melanoma. Atezolizumab, durvalumab, and avelumab are inhibitors targeting PD-L1. Atezolizumab is mainly utilized for treating NSCLC and urothelial carcinoma (27); durvalumab plays a critical role in the maintenance therapy following concurrent chemoradiotherapy in unresectable stage III NSCLC, as well as in NSCLC and urothelial carcinoma (28); avelumab is primarily indicated for the treatment of Merkel cell carcinoma and advanced urothelial carcinoma (29). Ipilimumab is the first CTLA-4 inhibitor approved for melanoma treatment, used in metastatic disease and as an adjuvant for stage III melanoma, enhancing T-cell activity by blocking the interaction between CTLA-4 and B7 molecules, thus promoting the immune system’s attack on tumor cells (30).

### 3.2 Mechanism of action

**Figure 3:**
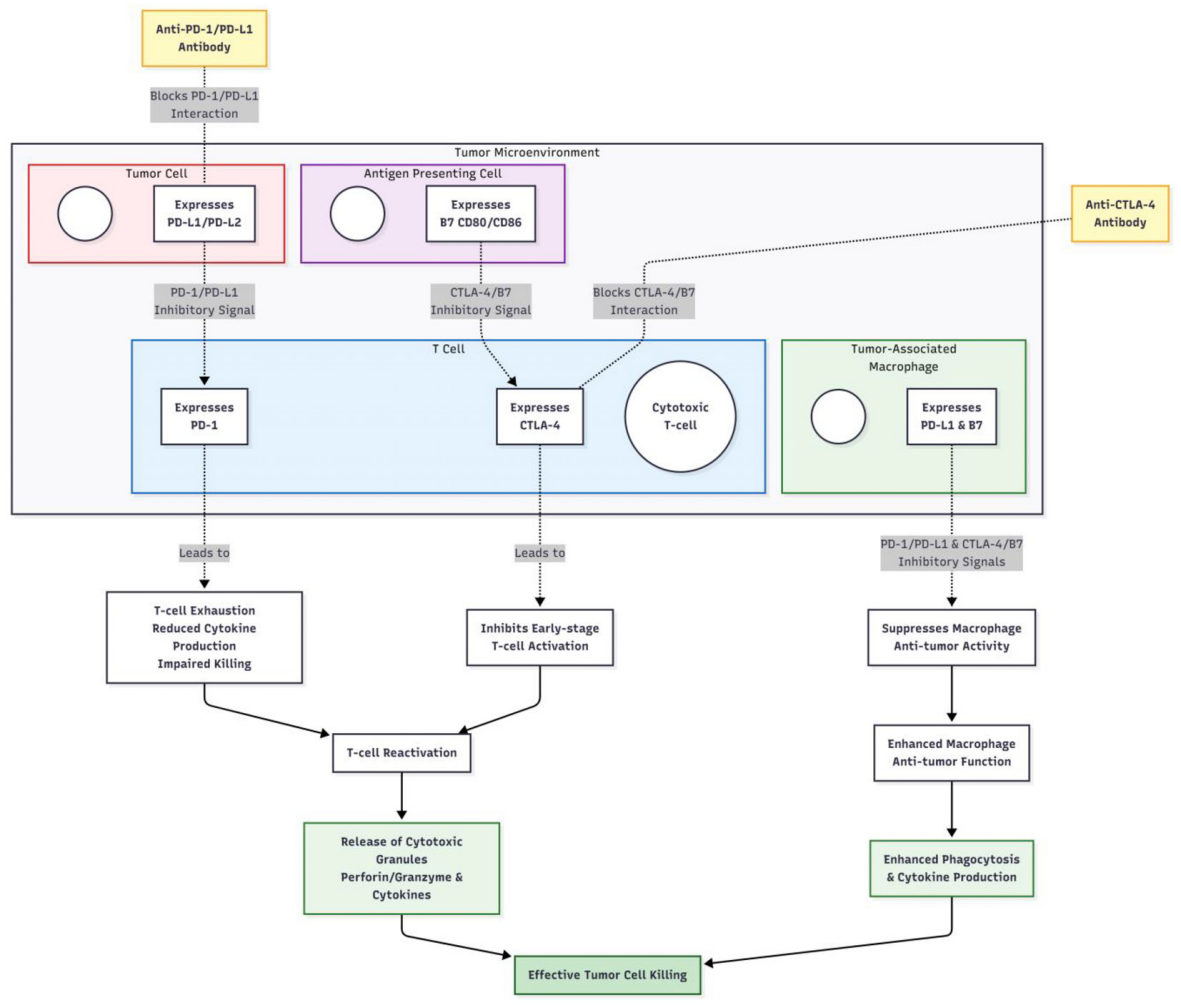
The mechanism of action of immune checkpoint inhibitors.

Under normal conditions, immune checkpoint proteins play an essential role in maintaining immunological homeostasis. For instance, the binding of PD-1 to its ligands PD-L1 and PD-L2 can inhibit T-cell activation, proliferation, and cytokine secretion, preventing self-tissue damage from excessive immune responses (31). Tumor cells can achieve immune evasion by overexpressing PD-L1, which binds to PD-1 on T cells, leading to suppression of T-cell function and ineffective recognition and killing of tumor cells (32). The mechanism of action of PD-1 inhibitors lies in blocking the interaction between PD-1 and its ligands PD-L1 and PD-L2, relieving the suppressed state of T cells and restoring their ability to kill tumor cells (33). CTLA-4 is predominantly expressed on activated T cells, and upon binding to the B7 molecule on the surface of antigen-presenting cells, it inhibits T-cell activation and proliferation. CTLA-4 inhibitors, such as ipilimumab, enhance T-cell activity and promote T-cell proliferation and cytokine secretion by blocking the binding of CTLA-4 to B7 molecules, thereby activating the immune system’s attack on tumor cells (34). Overall, immune checkpoint inhibitors disrupt the interaction between immune checkpoint proteins and their ligands, overcoming the immune evasion mechanisms employed by tumor cells, reactivating the body’s antitumor immune response, and enabling the immune system to effectively recognize and destroy tumor cells. The detailed cell-based mechanisms of antibody blockade and immune reactivation are illustrated in **Figure 3**. This mechanism is particularly critical in advanced (stage III/IV) melanoma, where T-cell exhaustion is prominent. In the adjuvant setting (stage III), ICIs aim to eliminate micrometastases and prevent recurrence.

## 4 Role of immune cells in melanoma treatment

This section discusses the roles of key immune cells in anti-melanoma immunity, with context provided for primary versus advanced disease settings where relevant. The functional status of these cells significantly influences the response to ICI therapy.

### 4.1 T cells

T cells play a central role in the body’s antitumor immune response, capable of specifically recognizing antigens on the surface of tumor cells and killing them. In the progression of melanoma, the functional status of T cells critically regulates tumor growth and metastasis. Upon recognition of melanoma cells by the immune system, naïve T cells are activated and differentiate into effector T cells, including cytotoxic T lymphocytes (CTLs) and helper T cells (Th). CTLs recognize and bind to tumor antigen peptides presented by major histocompatibility complex (MHC) class I molecules on the surface of tumor cells, leading to the direct lysis of these cells through the release of perforin, granzymes, and other cytotoxic substances (35). CTLs also secrete cytokines such as tumor necrosis factor-alpha (TNF-α), inducing apoptosis in tumor cells. Helper T cells enhance the activation, proliferation, and differentiation of CTLs via cytokine secretion, including interleukin-2 (IL-2), interferon-gamma (IFN-γ), among others, thereby strengthening the antitumor immune response (36). However, melanoma cells can suppress T cell function through various mechanisms to achieve immune evasion. For instance, high expression of immune checkpoint molecules like programmed death-ligand 1 (PD-L1) on tumor cells can bind to programmed death receptor 1 (PD-1) on T cells, inhibiting T cell activation, proliferation, and cytokine production, leading to T cell exhaustion (37). Regulatory T cells (Tregs) and myeloid-derived suppressor cells (MDSCs) within the tumor microenvironment can also inhibit T cell activity and function by secreting suppressive cytokines such as transforming growth factor-beta (TGF-β) and IL-10 (38). Moreover, factors within the tumor microenvironment, such as hypoxia and low pH levels, can influence T cell infiltration and functionality (39). Clinical research (40) has shown that immune checkpoint inhibitors can block PD-1/PD-L1 signaling pathways, releasing T cells from inhibition by tumor cells and reactivating their antitumor activity, thus significantly improving the treatment outcomes for melanoma patients. For example, PD-1 inhibitors such as pembrolizumab and nivolumab have achieved significant efficacy in melanoma treatment, prolonging patient survival (41). However, not all patients benefit from immune checkpoint inhibitor therapy, with some developing resistance. Therefore, gaining a deeper understanding of the mechanisms underlying T cell function in melanoma immunotherapy and strategies to overcome T cell suppression is crucial for enhancing treatment efficacy.

The efficacy of ICIs is fundamentally dependent on the functional reinvigoration of T cells. PD-1/PD-L1 blockade directly reverses the exhausted state of CD8^+^ cytotoxic T cells and CD4^+^ T helper cells within the TME, enhancing their cytokine production and cytotoxic capacity. Conversely, the abundance and suppressive function of regulatory T cells (Tregs) can limit ICI efficacy. Patients with a pre-existing CD8^+^ T cell infiltrate in tumors generally respond better to PD-1 blockade. In stage III-IV melanoma, lower levels of T cell exhaustion are associated with a 30-40% higher objective response rate to ICIs. Strategies to selectively target Tregs in combination with ICIs are under investigation (41). Thus, the composition and functional state of the T cell compartment are critical determinants of response to checkpoint blockade. The role of skin-resident memory T cells (TRM) is discussed in Section 4.1.

### 4.2 Natural killer cells

Natural killer (NK) cells are a vital component of the innate immune system, playing an indispensable role in antitumor immunity. Unlike T cells, NK cells can directly recognize and eliminate tumor cells without prior sensitization, exhibiting rapid responsiveness that enables them to perform immune surveillance during the early stages of tumorigenesis (42). The surface of NK cells expresses various activating and inhibitory receptors, which modulate NK cell activity through interactions with corresponding ligands on target cells (43). When the expression of major histocompatibility complex (MHC) class I molecules on tumor cells is downregulated or when certain abnormal ligands are expressed, the signaling from activating receptors on NK cells is enhanced while that from inhibitory receptors is diminished, leading to NK cell activation and their function in tumor cell killing (44). The mechanisms by which NK cells kill tumor cells include the following (45–47): Firstly, they release cytoplasmic granules containing perforin and granzymes; perforin forms pores in the target cell membrane, allowing granzymes to enter the target cell and activate caspase-dependent or independent apoptotic pathways, inducing apoptosis in tumor cells. Secondly, some NK cells express members of the tumor necrosis factor (TNF) family, such as FasL or TRAIL, which bind to the respective receptors Fas or TRAILR on target cells, initiating the apoptotic program in those cells. Thirdly, NK cells secrete multiple cytokines, including interferon-gamma (IFN-γ) and TNF-α, which inhibit tumor cell growth and proliferation and modulate the functions of other immune cells, thereby enhancing the body’s antitumor immune response.

In the microenvironment of melanoma, the functionality of NK cells is influenced by several factors. Cytokines secreted by tumor cells, such as transforming growth factor-beta (TGF-β) and interleukin-10 (IL-10), can suppress the activation and function of NK cells (48). Moreover, immunosuppressive cells associated with tumors, such as tumor-associated macrophages and myeloid-derived suppressor cells, can also interact with NK cells to diminish their antitumor activity (49). However, studies have found that certain immunomodulatory strategies, such as using cytokines like IL-2, IL-15 to activate NK cells, or combining immune checkpoint inhibitors, can enhance the role of NK cells in treating melanoma (50). Research has shown that the combination of IL-2 with adoptive NK cell therapy significantly improves clinical response rates in melanoma patients (51). IL-2 promotes the proliferation, activation, and survival of NK cells, enhancing their cytotoxic capability against tumor cells. Furthermore, immune checkpoint inhibitors may not only activate T cell function but also indirectly potentiate NK cell activity by modulating the tumor microenvironment. For example, PD-1 inhibitors could relieve inhibition on NK cells by blocking the PD-1/PD-L1 signaling pathway, thereby improving their antitumor effects (52). NK cells hold significant potential in the immunotherapy of melanoma, and exploring methods to enhance NK cell function may provide new strategies for its treatment.

While ICIs primarily target T cells, their success can be influenced by NK cell activity. NK cells contribute to antibody-dependent cellular cytotoxicity (ADCC), which might be relevant for certain antibody therapies. Furthermore, the inflammatory TME reshaped by effective ICI treatment may enhance NK cell recruitment and activation. However, resistance to ICIs can be associated with an inability to engage NK cells effectively. Combining IL-2 to activate NK cells has been shown to extend median overall survival by 4–6 months in some patients with stage IV melanoma (53), highlighting their potential as complementary players or targets in combination immunotherapy strategies (52).

### 4.3 Macrophages

Macrophages are highly heterogeneous and plastic immune cells that play a complex and multifaceted role in the development of melanoma and its immunotherapy. Depending on their microenvironment and functional state, macrophages can be classified into classically activated M1-type macrophages and alternatively activated M2-type macrophages (54). M1-type macrophages are primarily induced by stimuli such as IFN-γ and lipopolysaccharide (LPS), exhibiting potent antitumor activity (55). They secrete pro-inflammatory cytokines, including TNF-α, IL-1, IL-6, as well as cytotoxic substances like reactive oxygen species (ROS) and nitric oxide (NO), which directly kill tumor cells (56). Additionally, M1-type macrophages can present antigens to activate T cells, enhancing adaptive immune responses (57). M1-type macrophages also recruit other immune cells, such as NK cells and T cells, to the tumor site to participate in antitumor immune responses. In contrast, M2-type macrophages are mainly induced by stimuli like IL-4 and IL-13, with functions skewed towards promoting tumor growth, angiogenesis, and immunosuppression (58). M2-type macrophages secrete various growth factors, such as vascular endothelial growth factor (VEGF) and insulin-like growth factor (IGF), which promote tumor angiogenesis, supplying nutrients and oxygen to support tumor growth and metastasis (59). Moreover, M2-type macrophages can secrete immunosuppressive factors, including IL-10 and TGF-β, which inhibit the activity of immune cells such as T cells and NK cells, aiding tumor cells in evading immune surveillance (58).

In the tumor microenvironment of melanoma, macrophages predominantly exhibit an M2 phenotype, which is closely associated with poor prognosis (60). Tumor cells can recruit monocytes to the tumor site and induce their differentiation into M2-type macrophages by secreting various cytokines and chemokines such as CCL2 and CSF1 (61). Moreover, factors within the tumor microenvironment, including hypoxia and low pH levels, also promote the polarization of macrophages towards the M2 phenotype (62). However, recent studies (63) have shown that certain interventions can shift macrophage polarization towards the M1 phenotype, thereby enhancing their antitumor activity. For instance, small molecules and cytokines can be used to modulate the polarization state of macrophages. Research has found that Toll-like receptor 4 (TLR4) agonists can induce M1 polarization in macrophages, augmenting their cytotoxic capability against melanoma cells (64). Additionally, combining immune checkpoint inhibitors with macrophage modulators may represent an effective therapeutic strategy. By blocking immune checkpoint signaling pathways and simultaneously regulating macrophage polarization, it is possible to improve the efficacy of immunotherapy for melanoma.

The polarization state of TAMs significantly impacts ICI outcomes. A high M2/M1 ratio in the TME is generally associated with ICI resistance, as M2-TAMs promote immunosuppression. Patients with a high M2 macrophage infiltration have been associated with up to a 25% higher rate of resistance to ICIs (65). Strategies to reprogram M2-TAMs towards an M1-like, anti-tumor phenotype are being actively pursued to overcome resistance. For example, combining TLR4 agonists to induce M1 polarization with ICIs has been shown in preclinical and early clinical studies to potentially increase objective response rates by 15-20% (66). Additionally, some evidence suggests that ICIs themselves might indirectly affect macrophage function, and certain macrophage subsets express checkpoints like PD-L1, making them a direct target for antibody blockade (63).

### 4.4 T-helper cells

T-helper (Th) cells, a subset of CD4+ T cells, are crucial coordinators of adaptive anti-tumor immunity. Different Th cell subsets have distinct and often opposing roles in melanoma.

Th1 cells: These cells are characterized by the production of IFN-γ and IL-2. They promote the activation and cytotoxic function of CD8+ CTLs and enhance M1 macrophage polarization, fostering a pro-inflammatory, anti-tumor environment. A strong Th1 response is generally associated with better prognosis and improved response to ICIs (36).

Th2 cells: Th2 cells secrete IL-4, IL-5, and IL-13. They can promote B cell antibody class switching but also induce M2 macrophage polarization, which contributes to immunosuppression and tumor progression. A dominant Th2 response can inhibit effective anti-tumor immunity and is linked to poorer outcomes (58). The balance between Th1 and Th2 responses is critical. A high Th1/Th2 ratio is favorable for anti-tumor immunity. The Th1/Th2 balance is a potential biomarker for ICI response. Among stage III melanoma patients treated with PD−1 inhibitors, a higher peripheral−blood Th1/Th2 ratio (or a Th1−biased immune profile) is associated with improved long−term survival, whereas a lower Th1/Th2 ratio correlates with poorer outcomes (67). Therapeutic strategies aimed at inhibiting Th2 responses or boosting Th1 responses are being explored to overcome ICI resistance.

### 4.5 Dendritic cells

Dendritic cells (DCs) are the most potent professional antigen-presenting cells (APCs) and are essential for initiating and regulating T cell-mediated anti-tumor immunity. They capture tumor antigens, migrate to draining lymph nodes, and present processed peptides to naïve T cells, leading to their activation and differentiation into effector cells. In the skin, Langerhans cells are a specialized subset of DCs residing in the epidermis (see Section 4.1 for skin-specific roles). However, in the melanoma TME, DC function is often impaired. Tumor-derived factors such as IL-10, TGF-β, and VEGF can inhibit DC maturation, migration, and antigen-presentation capacity, leading to T cell tolerance instead of activation (68). The presence and functional state of DCs are critical for the success of ICIs, which rely on pre-existing T cell responses. Patients with a high density of mature DCs in their tumors have shown an approximately 20% higher objective response rate to ICIs. Strategies to enhance DC function, such as using FLT3 ligands or specific DC subsets like cDC1, are being explored to improve ICI responses. Furthermore, combining DC-based vaccines with ICIs has shown promise in re-sensitizing some ICI-resistant patients, by effectively priming new T cell responses (69).

### 4.6 B cells

B cells have emerged as important players in the anti-tumor immune response, beyond their classical role as antibody-producing cells. In melanoma, tumor-infiltrating B cells (TIBs) can be found within tertiary lymphoid structures (TLS), which are organized aggregates of immune cells that form in or near tumors (70). Within TLS, B cells can contribute to anti-tumor immunity by: (1) producing tumor-specific antibodies that may mediate antibody-dependent cellular phagocytosis (ADCP) or complement-dependent cytotoxicity (CDC); (2) acting as antigen-presenting cells to activate T cells; and (3) secreting immunostimulatory cytokines. The presence of TLS and TIBs is generally associated with improved patient survival and better response to ICIs in several cancer types, including melanoma. The presence of B cells and TLS serves as a positive prognostic marker for ICI response. In patients with stage IV melanoma, the presence of TIBs was associated with a median overall survival of 24 months after PD-1 inhibitor treatment, compared to 12 months in TIB-negative patients (71). However, certain regulatory B cell (Breg) subsets can also exert immunosuppressive effects via IL-10 secretion. Further research is needed to fully elucidate the multifaceted roles of B cells and to harness their potential for improving immunotherapy.

## 5 Influence of tumor microenvironment on melanoma and immunotherapy

### 5.1 Cellular composition

The skin microenvironment constitutes a complex ecosystem containing various cell types that interact with melanoma cells, collectively influencing tumor development and response to immunotherapy (72). Fibroblasts are important stromal cells within the skin microenvironment, playing a key role in the pathogenesis of melanoma (73). Cancer-associated fibroblasts (CAFs) can promote the proliferation, migration, and invasion of melanoma cells by secreting multiple cytokines and growth factors, such as transforming growth factor-beta (TGF-β), platelet-derived growth factor (PDGF), and vascular endothelial growth factor (VEGF) (74–76). TGF-β can inhibit immune cell activity and facilitate tumor immune evasion (77); PDGF stimulates the proliferation and activation of fibroblasts, affecting the remodeling of the tumor microenvironment (78); VEGF serves as a critical regulator of angiogenesis, promoting the formation of tumor vasculature to provide nutrients and oxygen to support tumor growth and metastasis (79). Furthermore, CAFs alter the physical properties of the tumor microenvironment through the secretion of extracellular matrix components, impacting tumor cell behavior (80).

Endothelial cells form the endothelial layer of blood vessels and play a central role in tumor angiogenesis. Melanoma cells secrete various angiogenic factors, such as VEGF and basic fibroblast growth factor (bFGF), which stimulate endothelial cell proliferation, migration, and lumen formation, thereby fostering the neovascularization of tumors (81). The newly formed tumor vasculature not only supplies necessary nutrients and oxygen to tumor cells but also provides a pathway for metastasis. Tumor vasculature exhibits structural and functional abnormalities, such as incomplete vessel walls and increased permeability, facilitating the entry of tumor cells into the circulation and distant metastasis (82). Additionally, endothelial cells can influence the immune status of the tumor microenvironment through interactions with immune cells. For example, endothelial cells express adhesion molecules like vascular cell adhesion molecule-1 (VCAM-1) and intercellular adhesion molecule-1 (ICAM-1), which promote immune cell adhesion and migration. However, their specific roles in the tumor microenvironment are complex and may either aid in the recruitment of immune cells to the tumor site or contribute to mechanisms of tumor immune evasion (83).

The skin microenvironment harbors unique immune populations with distinct roles in melanoma immunosurveillance and pathology. Tissue-resident memory T cells (TRM): These are long-lived T cells that persist in the skin after antigen exposure and do not recirculate. They provide rapid frontline defense against local pathogen or tumor recurrence. In cutaneous melanoma, the presence of CD8^+^ TRM cells (expressing markers like CD69 and CD103) in the tumor is associated with improved prognosis and better response to ICIs. A study (84) showed that in patients with stage III cutaneous melanoma, a high density of TRM cells was associated with an 18% reduction in recurrence rate following adjuvant ICI therapy. Langerhans Cells (LCs): These are the specialized dendritic cells residing in the epidermal layer of the skin. They are the first antigen-presenting cells encountered by cutaneous melanoma cells (85). LCs capture tumor antigens and migrate to draining lymph nodes to initiate T cell responses. However, in melanoma, the number and function of LCs can be impaired. Their density is often decreased in melanoma lesions, potentially contributing to defective antigen presentation and immune tolerance (86). Keratinocyte-Immune Crosstalk: Keratinocytes, the primary cells of the epidermis, are active participants in skin immunity. They can secrete cytokines and chemokines that shape the immune landscape (87). For instance, keratinocyte-derived IL-18 can promote NK cell activation, enhancing the clearance of early melanoma cells. Conversely, in the tumor-promoting microenvironment, keratinocytes may be induced to secrete immunosuppressive factors like IL-10, which can inhibit NK and T cell function. This crosstalk highlights the integrated nature of the cutaneous TME.

Additionally, the skin microenvironment includes other cellular components such as mast cells and dendritic cells (the general role of DCs is covered in Section 3.5). Mast cells can influence tumor cell growth and immune cell function by releasing mediators such as histamine and cytokines (88). Activation of mast cells within the tumor microenvironment may be associated with tumor progression and immune evasion (89).

### 5.2 Extracellular matrix

The extracellular matrix (ECM) is a critical component of the skin microenvironment, composed of multiple proteins such as collagen, elastin, fibronectin, laminin, and polysaccharides like glycosaminoglycans (90). The ECM not only provides physical support for cells but also plays a significant regulatory role in biological processes such as cell proliferation, differentiation, migration, and adhesion (91). During the development of melanoma, the composition and structure of the ECM undergo substantial changes (92). Tumor cells secrete various proteases, such as matrix metalloproteinases (MMPs), which degrade ECM components and disrupt its normal architecture, facilitating tumor cell invasion and metastasis. MMPs can break down ECM proteins like collagen and fibronectin, allowing tumor cells to more easily penetrate the basement membrane and invade surrounding tissues. Moreover, tumor cells can regulate ECM synthesis and remodeling through the secretion of cytokines and growth factors, creating a microenvironment conducive to tumor cell survival and proliferation (93). The ECM can also interact with receptors on the surface of tumor cells to modulate intracellular signaling pathways. Integrins, a class of transmembrane receptors widely expressed on cell surfaces, bind to various ECM components such as collagen, fibronectin, and laminin (94). The interaction between integrins and the ECM not only mediates cell adhesion but also activates intracellular signaling pathways, including mitogen-activated protein kinase (MAPK) and phosphoinositide 3-kinase (PI3K)/protein kinase B (AKT) pathways, regulating tumor cell proliferation, survival, migration, and invasion (95). In melanoma, aberrant expression of certain integrin subtypes has been linked to tumor progression and metastasis. Research has found that high expression of some integrin subtypes can enhance the adhesion, migration, and invasive capabilities of melanoma cells, increasing their metastatic potential (96). Furthermore, the ECM influences the infiltration and function of immune cells within the tumor microenvironment. The physical properties of the ECM, such as stiffness and elasticity, can impact immune cell migration and localization (97). A stiffer ECM may impede the infiltration of immune cells into the tumor site, whereas appropriate ECM remodeling can facilitate the recruitment and activation of immune cells (98). Components of the ECM, such as fibronectin and laminin, can also interact with receptors on immune cells to modulate their activity and function (99). Fibronectin can promote T cell adhesion and activation, enhancing antitumor immune responses, while laminin may have inhibitory effects on immune cell function. Therefore, understanding the mechanisms of interaction between the ECM and immune cells is crucial for optimizing immunotherapy strategies for melanoma. In advanced (stage IV) melanoma, increased ECM stiffness is a common feature that can act as a physical barrier to T cell infiltration, contributing to ICI resistance; studies suggest this may be associated with up to a 30% higher rate of resistance to PD-1 inhibitors (92).

### 5.3 Cytokines and chemokines

Cytokines and chemokines are critical signaling molecules within the skin microenvironment, playing a key role in regulating the recruitment, activation, and function of immune cells (100). Cytokines are small protein molecules secreted by immune cells and other cell types, including interleukins (ILs), interferons (IFNs), tumor necrosis factors (TNFs), among others (101). In the tumor microenvironment of melanoma, the cytokine expression profile undergoes significant changes that can influence tumor cell growth, immune evasion, and the efficacy of immunotherapy. Interleukin-6 (IL-6) is a multifunctional cytokine that is frequently overexpressed in the tumor microenvironment of melanoma (102). IL-6 can promote the proliferation, survival, and invasiveness of melanoma cells by activating the signal transducer and activator of transcription 3 (STAT3) signaling pathway. Additionally, IL-6 can inhibit T cell activation and proliferation and facilitate the differentiation and expansion of regulatory T cells (Tregs), thereby suppressing antitumor immune responses. Interleukin-10 (IL-10) is an important immunosuppressive cytokine that can inhibit the function of antigen-presenting cells such as macrophages and dendritic cells, reduce the secretion of pro-inflammatory cytokines, and lower the activity of T cells and natural killer (NK) cells, aiding tumor cells in evading immune surveillance (103). Interferon-gamma (IFN-γ) is a cytokine with potent immunomodulatory and antitumor properties. IFN-γ can promote the polarization of macrophages towards the M1 phenotype, enhancing their antitumor activity (104), and it can also upregulate the expression of major histocompatibility complex (MHC) class I molecules on the surface of tumor cells, improving the efficiency of antigen presentation and the recognition and killing of tumor cells by T cells (105). However, in the tumor microenvironment of melanoma, the function of IFN-γ may be suppressed, with tumor cells employing multiple mechanisms to resist its effects and achieve immune evasion (106).

Chemokines are small protein molecules capable of attracting immune cells to migrate directionally; they guide the accumulation of immune cells at specific sites through interactions with chemokine receptors on the surface of immune cells. Dysregulated expression of chemokines and their receptors in the tumor microenvironment of melanoma is closely associated with tumor progression and immune evasion. C-C motif chemokine ligand 2 (CCL2) is an important chemokine that recruits immune cells such as monocytes and macrophages to the tumor site (107). In melanoma, tumor cells secrete substantial amounts of CCL2, attracting monocytes and inducing their differentiation into M2-type macrophages, which promote tumor growth and immunosuppression. The C-X-C motif chemokine ligand 12 (CXCL12) and its receptor CXCR4 play a crucial role in the metastasis of melanoma (108). CXCL12, primarily secreted by stromal cells within the tumor microenvironment, binds to CXCR4 on the surface of melanoma cells, activating downstream signaling pathways that enhance tumor cell migration, invasion, and metastasis (109). Moreover, CXCL12 can modulate the recruitment and function of immune cells, affecting the immune state of the tumor microenvironment. Studies have shown that activation of the CXCL12/CXCR4 axis can inhibit T cell infiltration and function, promoting immune evasion by tumor cells (110). In cutaneous melanoma, the skin-specific chemokine CCL27 plays a role in recruiting skin-homing T cells, including TRM cells, to the site of disease. High expression of CCL27 in primary cutaneous melanomas is associated with enhanced T cell infiltration and has been linked to a 25% improvement in local tumor control rates following ICI therapy (111). In summary, cytokines and chemokines form a complex network within the tumor microenvironment of melanoma, influencing tumor cell growth, immune evasion, and the outcomes of immunotherapy by regulating the recruitment, activation, and function of immune cells. A deeper understanding of the mechanisms by which cytokines and chemokines contribute to the pathogenesis of melanoma could provide theoretical foundations for developing new therapeutic targets and strategies.

### 5.4 Skin microbiome

The skin microbiome, the diverse community of commensal microorganisms residing on the skin, is increasingly recognized as a modulator of local and systemic immunity, potentially influencing melanoma development and response to therapy. The composition of the skin microbiome, typically dominated by genera such as Staphylococcus and Cutibacterium, can shape the immune landscape of the skin. Certain commensal bacteria can enhance anti-tumor immunity by activating pattern recognition receptors (e.g., Toll-like receptors TLR2) on skin immune cells, promoting a pro-inflammatory state that may prime the immune system for better response to ICIs. Conversely, dysbiosis, an imbalance in the microbial community, characterized by an overgrowth of potentially pathogenic species like Staphylococcus aureus, can promote an immunosuppressive environment. These pathogens may secrete factors that inhibit T cell function or promote the expansion of regulatory immune cells, thereby diminishing the efficacy of ICIs. Clinical evidence is emerging to support this link. A study (112) reported that patients with stage III melanoma who had a higher diversity of the skin microbiome experienced an objective response rate of 58% to PD-1 inhibitors, significantly higher than the 32% response rate observed in patients with low microbiome diversity. This highlights the potential of the skin microbiome as a predictive biomarker and a therapeutic target, with strategies like topical probiotics or prebiotics being explored to modulate the microbiome for clinical benefit.

### 5.5 Metabolites in tumor microenvironment

The metabolic landscape of the TME is a key regulator of immune cell function and a contributor to ICI resistance. Tumor cells and stromal cells undergo metabolic reprogramming, leading to the accumulation of metabolites that can directly suppress anti-tumor immune responses. Key immunosuppressive metabolites include:

Lactate: A byproduct of aerobic glycolysis (the Warburg effect) highly produced by tumor cells. Lactate acidifies the TME and directly inhibits the function and cytokine production of T cells and NK cells (113).

Kynurenine: Generated from tryptophan metabolism by enzymes like indoleamine 2,3-dioxygenase 1 (IDO1), which is often upregulated in melanoma. Kynurenine promotes the differentiation of regulatory T cells (Tregs) and induces T cell apoptosis, contributing to immune tolerance (114).

Targeting these metabolic pathways is a promising strategy to overcome ICI resistance. For instance, inhibiting lactate production (e.g., via lactate dehydrogenase inhibitors) or blocking the kynurenine pathway (e.g., with IDO1 inhibitors) can reverse T cell suppression and enhance ICI efficacy in preclinical models. Early-phase clinical trials combining IDO1 inhibitors with ICIs have shown mixed results, underscoring the complexity of metabolic targeting. However, a study (115) suggested that combining a lactate dehydrogenase inhibitor with anti-PD-1 therapy could increase the objective response rate by approximately 15% in a subset of ICI-resistant patients. The accumulation of these metabolites often increases with disease progression. For example, lactate levels are significantly higher in stage IV metastatic melanoma compared to stage III disease, which may partly explain the higher rates of ICI resistance observed in advanced metastatic settings.

## 6 Clinical application and challenges of immune checkpoint inhibitors in melanoma therapy

### 6.1 Clinical efficacy of ICIs across disease stages

Immune checkpoint inhibitors have demonstrated significant efficacy across the spectrum of melanoma, from metastatic disease to the adjuvant and neoadjuvant settings. Robust evidence from multiple clinical trials supports their use.

#### 6.1.1 Metastatic melanoma (stage IV)

In advanced, unresectable stage IV melanoma, ICIs have fundamentally improved survival outcomes. The CheckMate 067 trial (116), a landmark phase III study, compared the combination of nivolumab and ipilimumab versus nivolumab monotherapy versus ipilimumab monotherapy. The results demonstrated a median overall survival (OS) of 71.9 months for the combination group, significantly longer than the 36.9 months for nivolumab monotherapy and 19.9 months for ipilimumab monotherapy. The objective response rate (ORR) was 58% for the combination, compared to 44% for nivolumab and 19% for ipilimumab. The KEYNOTE-006 study (117) evaluated pembrolizumab versus ipilimumab, showing a three-year OS rate of 44.2% for pembrolizumab versus 32.0% for ipilimumab, with ORRs of 33.7% and 11.9%, respectively. These findings established the superiority of anti-PD-1 based therapies over CTLA-4 inhibition and confirmed the potent, albeit more toxic, efficacy of combination therapy in metastatic disease.

#### 6.1.2 Adjuvant therapy (stage III)

Adjuvant therapy with ICIs aims to eliminate micrometastatic disease after complete surgical resection of high-risk melanoma, thereby reducing the risk of recurrence. Several pivotal trials have led to approvals in this setting:

CheckMate 238 (118):This trial compared nivolumab to ipilimumab (10 mg/kg) in patients with resected stage IIIB-IV melanoma. Nivolumab demonstrated superior recurrence-free survival (RFS) with a lower rate of high-grade adverse events. The 2-year RFS rates were 62% for nivolumab versus 52% for ipilimumab.

KEYNOTE-054 (EORTC 1325) (119): This study evaluated pembrolizumab versus placebo in patients with resected stage IIIA-IIIC melanoma. It showed a significant improvement in RFS for the pembrolizumab group, with a 3-year RFS of 65.4% in the PD-L1 positive population versus 45.1% for placebo.

EORTC 18071 (120): This earlier trial established the role of CTLA-4 blockade, showing that adjuvant ipilimumab (10 mg/kg) improved RFS and OS compared to placebo in stage III patients, albeit with significant toxicity. The 5-year RFS was 26% for ipilimumab versus 17% for placebo. The IMMUNED trial further investigated the combination of ipilimumab and nivolumab in the adjuvant setting, showing high efficacy but also a very high rate of severe toxicity, limiting its routine use.

#### 6.1.3 Neoadjuvant therapy (resectable stage III)

Neoadjuvant ICI therapy, administered before surgery for resectable stage III melanoma, has emerged as a highly promising approach. It aims to induce a robust anti-tumor immune response early, potentially leading to higher pathological response rates and improved long-term outcomes. Key trials include:

SWOG S1801 (121): This phase II trial compared neoadjuvant pembrolizumab followed by adjuvant pembrolizumab to adjuvant pembrolizumab alone in patients with resectable stage IIIB-IV melanoma. The neoadjuvant approach significantly improved event-free survival. These studies explored different dosing schedules of neoadjuvant ipilimumab plus nivolumab in stage III melanoma. OpACIN-neo identified a regimen with improved safety and a high pathological response rate, including pathological complete responses (pCR) in a substantial proportion of patients.

An extension of the OpACIN-neo platform, the PRADO study investigated a response-guided approach following neoadjuvant ipilimumab+nivolumab, allowing for personalized de-escalation of surgery based on the degree of pathological response (122). These studies collectively suggest that neoadjuvant ICI can achieve PCR rates of 25-40% or more, which is associated with excellent long-term survival. It is important to note that the majority of the landmark trials cited here primarily enrolled patients with cutaneous melanoma; efficacy in mucosal or acral subtypes requires further validation.

### 6.2 Adverse reactions

Despite the significant efficacy of immune checkpoint inhibitors in melanoma, they are associated with a range of immune-related adverse events (irAEs). irAEs can affect multiple organs and tissues throughout the body, commonly involving the skin, gastrointestinal tract, endocrine glands, liver, and lungs (123). Skin reactions represent one of the most frequent irAEs, manifesting as rash, pruritus, vitiligo, and other conditions (124–126). The incidence of skin adverse reactions in patients receiving immune checkpoint inhibitors can reach 34%-43% (127). Vitiligo is particularly noted in melanoma patients and should be differentiated from other potentially life-threatening conditions such as drug-induced hypersensitivity syndrome with eosinophilia and systemic symptoms, Sweet’s syndrome, Stevens-Johnson syndrome, and toxic epidermal necrolysis (128). Gastrointestinal adverse reactions primarily consist of colitis and diarrhea. For CTLA-4 inhibitors, the peak incidence of diarrhea and colitis typically occurs around week 8 of treatment (129). Severe gastrointestinal adverse reactions can lead to dehydration and electrolyte disturbances, impacting patient quality of life and adherence to treatment (130). Endocrine irAEs can manifest as hypothyroidism, hyperthyroidism, and hypophysitis, among others. For instance, the risk of hypophysitis increases from week 7 following ipilimumab treatment and remains elevated throughout the course of treatment (131). Hepatotoxicity, another common irAE, can present as elevated transaminases and jaundice (132). The incidence of hepatotoxicity varies depending on the agent and regimen used; anti-PD-1 monoclonal antibodies tend to show sustained higher levels of hepatotoxicity between weeks 8 and 14 (133). Overall irAE occurrence ranges from 54% to 76%, with lower incidences of grade 3/4 toxicities (134). The frequency of irAEs is higher with CTLA-4 inhibitors compared to PD-1/PD-L1 inhibitors, which have similar rates of irAEs (135). Combination therapies result in higher irAE incidence, with the 3–4 grade irAE incidence approaching 40% for the combination of nivolumab and ipilimumab (136). The incidence and severity of irAEs can also vary by treatment setting. In the adjuvant setting (stage III), where treatment doses may be standardized and patients are generally healthier post-resection, the incidence of grade 3/4 irAEs is typically lower (e.g., 35-45% for combination therapy, 10-20% for anti-PD-1 monotherapy (137)] compared to the metastatic setting [stage IV, 50-60% for combination, 15-25% for anti-PD-1 monotherapy (138)), where higher tumor burden and patient frailty may contribute. Management of irAEs should adhere to principles of early prevention, continuous monitoring, and follow-up. Prior to treatment, clinicians must assess the patient’s susceptibility to irAEs and conduct relevant tests. Upon occurrence of irAEs, accurate diagnosis and severity assessment should guide stratified management. Most irAEs can be controlled through dose interruption or administration of corticosteroids and are reversible. For more severe adverse events, discontinuation of immune checkpoint inhibitor therapy may be necessary, with consultation from specialty physicians and potential hospitalization considered.

### 6.3 Resistance issues

Despite the significant survival benefits that immune checkpoint inhibitors provide to melanoma patients, resistance remains a substantial challenge in clinical treatment. Approximately 55% of melanoma patients exhibit intrinsic resistance to monotherapy with PD-1 inhibitors, and around 40% show intrinsic resistance to the combination of CTLA-4 and PD-1 inhibitors (139). Nearly 25% of responding patients develop resistance to PD-1 inhibitors within two years post-treatment (140). The mechanisms underlying resistance are complex and multifaceted (141–143), including:

1. Changes in PD-L1 expression on tumor cells, which can evade the effects of immune checkpoint inhibitors through upregulation or downregulation.
2. Deficiency in tumor antigens or ineffective antigen presentation, preventing T cells from recognizing tumor cells effectively. For instance, deficiencies in β2-microglobulin (β2M) and human leukocyte antigen (HLA) antigen presentation mechanisms can allow tumor cells to escape antigen recognition and presentation.
3. Activation of oncogenic pathways such as the PI3K/AKT pathway, WNT/β-catenin pathway, JAK/STAT/IFNγ pathway, and MAPK pathway, which may lead to resistance to immunotherapy due to aberrant signaling.
4. The role of immunosuppressive cells in the tumor microenvironment, including regulatory T cells (Tregs), myeloid-derived suppressor cells (MDSCs), and tumor-associated macrophages (TAMs), which can inhibit T cell activity and promote immune evasion by tumor cells.

Emerging mechanisms related to the TME, as discussed in this review, include skin microbiome dysbiosis leading to suppressed T cell function, increased ECM stiffness acting as a physical barrier to immune cell infiltration, and the accumulation of immunosuppressive metabolites like lactate and kynurenine.

To address resistance, researchers are exploring multiple strategies. Combination therapies represent one important approach, such as combining immune checkpoint inhibitors with other targeted agents, chemotherapy, radiation therapy, or cancer vaccines. A study (144) demonstrated that combining anti-PD-1 therapy with small molecule inhibitors like sunitinib could deplete mast cells, leading to complete regression of tumors in mice and prolonged survival. Enhancing the activity of immune cells by modulating the tumor microenvironment is also considered an effective way to overcome resistance. For example, using small molecules or cytokines to regulate the polarization state of macrophages, promoting their conversion to the M1 phenotype with antitumor activity. Novel strategies targeting the newly described mechanisms are under investigation, including combining ICIs with skin microbiome modulation (e.g., topical probiotics), ECM-remodeling agents (e.g., MMP inhibitors), or drugs targeting metabolic pathways (e.g., lactate dehydrogenase inhibitors). Understanding the mechanisms of resistance and developing effective countermeasures are crucial for improving long-term survival rates and quality of life for melanoma patients.

## 7 Conclusion and future perspectives

### 7.1 Summary of research

The advent of immune checkpoint inhibitors has revolutionized the treatment of melanoma, significantly improving patient survival and quality of life. Their mechanism of action primarily involves blocking interactions between immune checkpoint proteins and their ligands, thereby releasing the inhibition exerted by tumor cells on the immune system and reactivating the body’s antitumor immune response. However, challenges persist in treating melanoma with immune checkpoint inhibitors, including non-responsive patients, resistance development, and immune-related adverse events. Immune cells play a central role in the immunotherapy of melanoma, with T cells, natural killer cells, macrophages, T helper cells, dendritic cells, and B cells participating in antitumor immune responses via distinct mechanisms. Nevertheless, tumor cells can suppress immune cell function in various ways, leading to immune evasion. The tumor microenvironment, as a critical site for tumor growth and survival, profoundly influences immune cell function and activity due to its complex composition and unique physicochemical properties, closely linking it to the development of melanoma and the efficacy of immunotherapy. This review has highlighted the application of ICIs across different disease stages (primary, adjuvant, neoadjuvant, metastatic) and emphasized the roles of skin-specific immune components (e.g., TRM, Langerhans cells) and microenvironmental factors (e.g., skin microbiome, metabolites). There is a complex interplay between immune cells and the TME; immune cells can modulate the TME through cytokine secretion, while the TME can affect immune cell infiltration, activation, and function. A deeper understanding of these interactions is essential for optimizing immunotherapy strategies for melanoma.

### 7.2 Directions for future research

Future research on immunotherapy for melanoma will focus on several key areas:

1. Exploring combination therapy regimens, such as pairing immune checkpoint inhibitors with other targeted drugs, chemotherapy, radiation therapy, cancer vaccines, or cell therapies, to enhance therapeutic efficacy and overcome resistance. This includes rational combinations based on TME modulation, such as ICIs with angiogenesis normalizers, macrophage polarizing agents, or metabolic inhibitors.
2. Strengthening biomarker research to identify markers that accurately predict the effectiveness of immune checkpoint inhibitor therapy, enabling precision medicine approaches to select patients who are more likely to benefit from treatment while minimizing unnecessary treatments and adverse reactions. Beyond PD-L1 and TMB, promising biomarkers include the gut and skin microbiome, T cell clonality, specific TME features (e.g., TLS presence), and circulating tumor DNA (ctDNA).
3. Deepening the study of the tumor microenvironment, especially the regulation of the skin and systemic TME, to develop therapeutic strategies targeting this environment, ameliorate immunosuppression, and boost the antitumor activity of immune cells. Specific future directions include: Exploring the precise mechanisms linking the skin microbiome to ICI efficacy and developing personalized microbiome-modulating interventions. Designing targeted therapies to enhance the function of skin-resident immune cells like TRM for improved local control of cutaneous melanoma. Conducting dedicated clinical trials for mucosal and acral melanoma subtypes to address the current gap in evidence for ICIs in these settings.
4. Conducting personalized treatment strategy research, tailoring individualized treatment plans based on patients’ genetic characteristics, features of the tumor microenvironment, and immune status, to increase the specificity and effectiveness of therapy.

As our understanding of the pathogenesis of melanoma and immunotherapy continues to deepen, along with the emergence of new technologies and drugs, we anticipate that melanoma treatment will achieve more significant progress, offering patients greater hope for survival and improved quality of life.
